# Supplementary material for: Biomimetic Oil-in-Water Nanoemulsions as a Suitable Drug Delivery System to Target Inflamed Endothelial Cells
Source: Nanomaterials (Basel). 2024 Jul 31;14(15):1286. doi: 10.3390/nano14151286 (PMC11314564; doi:10.3390/nano14151286)
Supplement: Supplementary file 1 [file nanomaterials-14-01286-s001.zip › nanomaterials-3079991-supplementary.pdf]

Supporting Information (SI).

Immunofluorescence (IF)

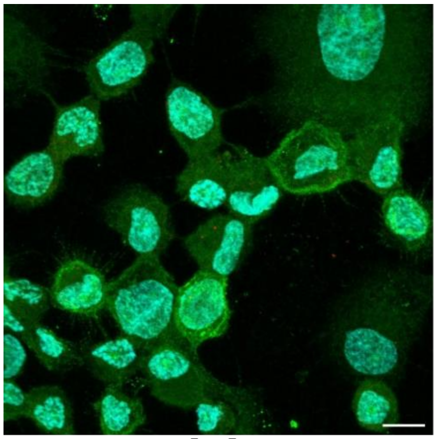

(a)

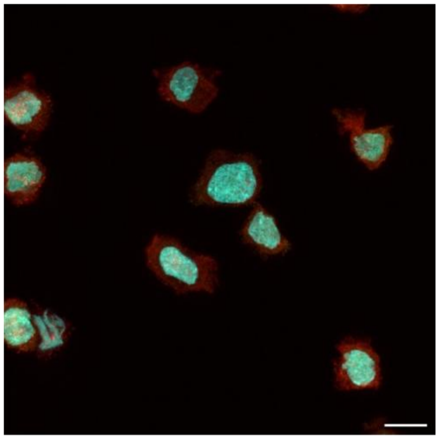

(b)

Figure S1 a) THP-1 PMA 16h activated macrophage cells green signal for CD55 expression **b)** THP-1 not PMA treated cells (monocytes) red signal for CD33 expression.

Membrane extraction and characterisation

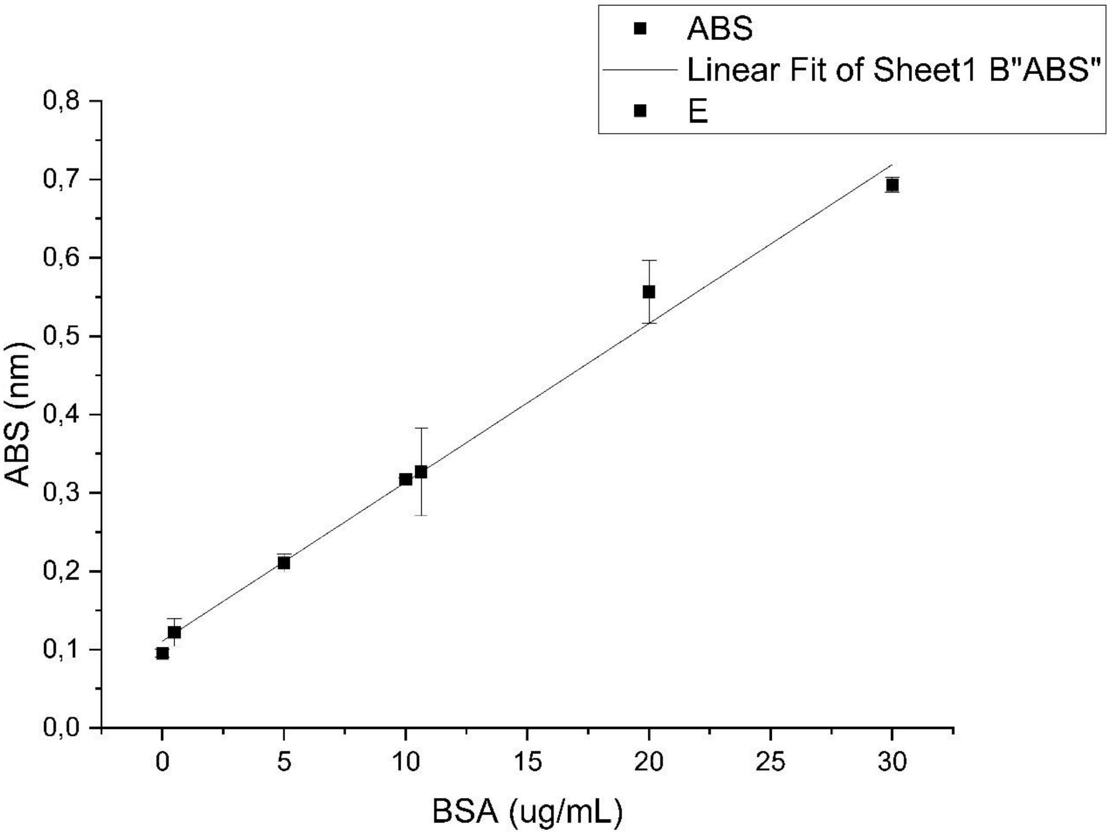

Figure S2. BCA calibration curve.

Table S1. Data from deconvolution analysis of CD spectra for isolated plasma membrane of monocytes and macrophages.

Sample	α-helix (%)	β-sheet (%)	Random (%)
**Monocytes**	40.7	15.0	44.3
**Macrophages**	36.5	16.3	47.2

Biomimetic NEsoSome development

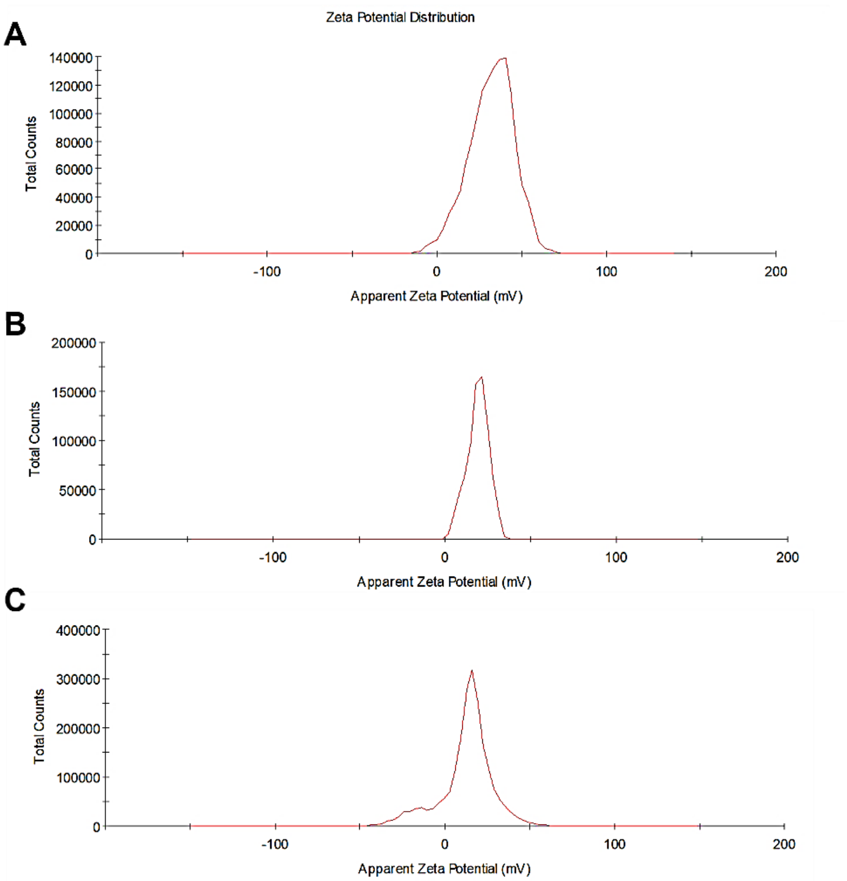

Figure S3. ζ-potential distributions of **a)** NEsoSome, **b)** M-NEsoSome and **c)** M0-NEsoSome.

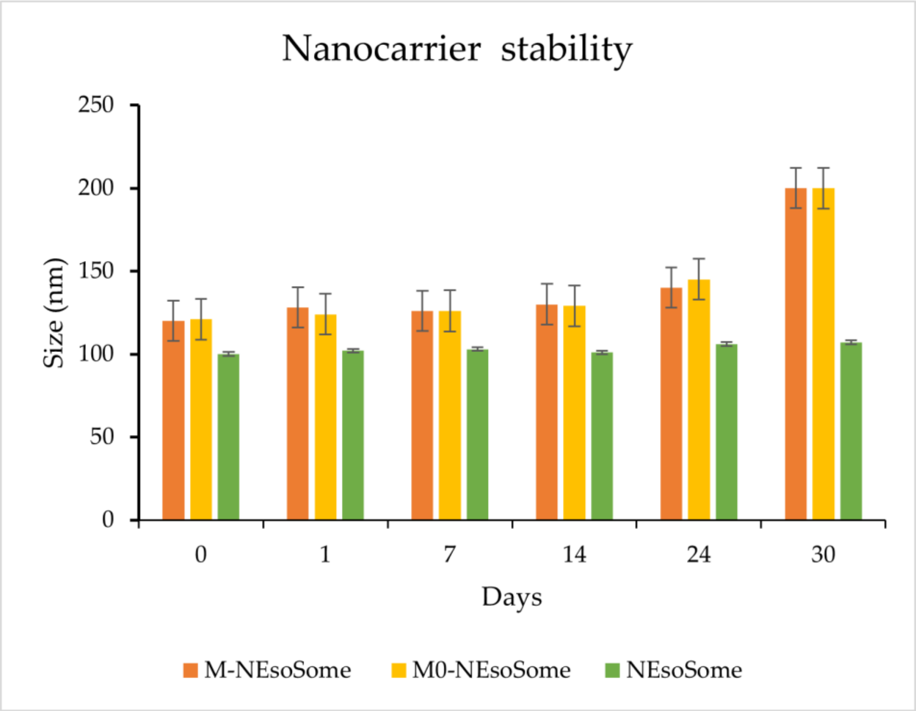

Figure S4 Dimensional stability over the time of NEsoSome, M-NEsoSome and M0-NesoSome. Data are reported as mean (n=3) ± SD.

In vitro accumulation analysis in HUVECs

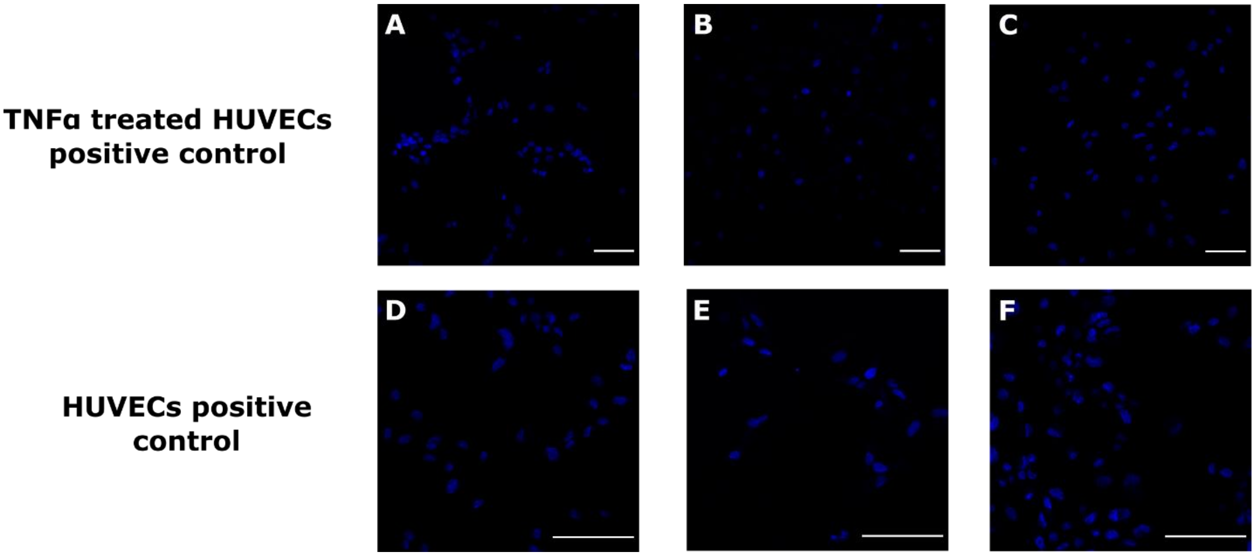

Figure S5. Positive control (treatment with cell medium) at different time points of TNF*α* treated HUVECs (**a**, **b**, **c**) and healthy HUVECs (**d**, **e**, **f**).

A

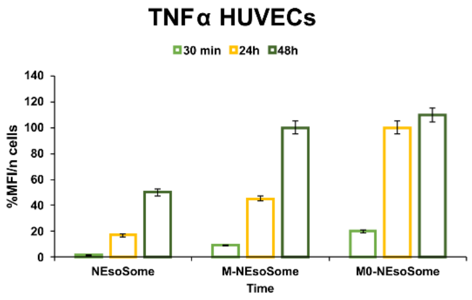

B

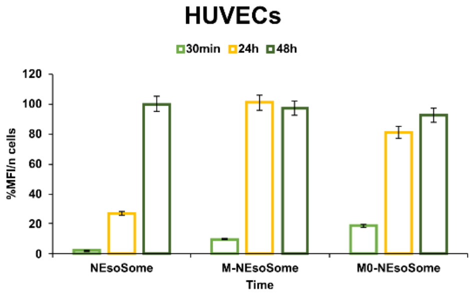

Figure S6. Plot of mean fluorescence intensity of nanocarrier normalised to cell number. TNFα HUVECs (**a**) and HUVECs (**b**) were treated with NEsoSome, M-NEsoSome and M0-NesoSome at different time points: 30min, 24h and 48h. Data are reported as mean (n=5) ± SD.
